# The effects of habitat management on the species, phylogenetic and functional diversity of bees are modified by the environmental context

**DOI:** 10.1002/ece3.1963

**Published:** 2016-01-18

**Authors:** Markus A. K. Sydenham, Stein R. Moe, Diana N. Stanescu‐Yadav, Ørjan Totland, Katrine Eldegard

**Affiliations:** ^1^ Department of Ecology and Natural Resource ManagementNorwegian University of Life SciencesP.O.B. 5003NO‐1432ÅsNorway

**Keywords:** Bees, biodiversity, functional diversity, habitat management, phylogenetic diversity, pollinators, power line clearings

## Abstract

Anthropogenic landscape elements, such as roadsides, hedgerows, field edges, and power line clearings, can be managed to provide important habitats for wild bees. However, the effects of habitat improvement schemes in power line clearings on components of diversity are poorly studied. We conducted a large‐scale experiment to test the effects of different management practices on the species, phylogenetic, and functional diversity of wild bees in power line clearings (*n* = 19 sites across southeastern Norway) and explored whether any treatment effects were modified by the environmental context. At each site, we conducted the following treatments: (1) Cut: all trees cut and left to decay in the clearing; (2) Cut + Remove: all trees cut and removed from the plot; and (3) Uncut: uncleared. The site‐specific environmental context (i.e., elevation and floral diversity) influenced the species, phylogenetic, and functional diversity within bee species assemblages. The largest number of species was found in the Cut + Remove treatment in plots with a high forb species richness, indicating that the outcome of management practices depends on the environmental context. Clearing of treatment plots with many forb species also appeared to alter the phylogenetic composition of bee species assemblages, that is, more closely related species were found in the Cut and the Cut + Remove plots than in the Uncut plots. *Synthesis and applications*: Our experimental simulation of management practices in power line clearings influenced the species, phylogenetic, and functional diversity of bee species assemblages. Frequent clearing and removal of the woody debris at low elevations with a high forb species richness can increase the value of power line clearings for solitary bees. It is therefore important for managers to consider the environmental context when designing habitat improvement schemes for solitary bees.

## Introduction

Human disturbance is currently reducing biodiversity globally (Dirzo et al. 2014). Declining populations of animal pollinators (Potts et al. 2010) are of particular concern, as they sustain sexual reproduction of an estimated 88% of wild angiosperm species (Ollerton et al. 2011). The bees (Hymenoptera: Apiformes) is an important group of pollinators, and intensified agriculture and other land use changes during the past century have caused increased extinction rates of wild bees (Ollerton et al. 2014) and declines in regional population densities and occurrences (Biesmeijer et al. 2006). Currently, about 9% of the European bee species are threatened (Nieto et al. 2014). The availability of suitable habitats is a limiting factor for bees in modern agricultural landscapes, and a positive relationship between the proportion of semi‐natural habitats and bee diversity in these landscapes has been reported in a number of studies (Steffan‐Dewenter et al. 2002; Winfree et al. 2011).

Semi‐natural grasslands, such as calcareous grasslands, provide important habitats for bees (Murray et al. 2012), but are often lost due to changes in land use, particularly reduced livestock grazing (Stoate et al. 2009). However, other anthropogenic landscape elements, such as power line clearings (Russell et al. 2005), hedgerows (Morandin and Kremen 2013), and agricultural field edges (Sydenham et al. 2014), may also provide important habitats for bees in the agricultural landscape matrix. For example, road verges may mimic semi‐natural habitats if re‐sown with native plant species (Hopwood 2008) or managed to promote the species richness of forbs (Noordijk et al. 2009). In addition, restoring hedgerows along fields increases the occurrence of specialized bees, which typically decline in richness and density in agricultural landscapes (Kremen and M'Gonigle 2015). Thus, developing ecologically sound management plans for marginal areas is of high importance for bee conservation (Nieto et al. 2014). However, as the response of bees to habitat improvement schemes in agricultural landscapes may depend on the initial quality of the habitat (Scheper et al. 2013), the outcome of management practices should be assessed under different environmental contexts before widely implemented.

In many forested landscapes, the establishment and maintenance of power line clearings have created extensive networks of habitat of early successional vegetation (Wojcik and Buchmann 2012). In Norway, where our study is conducted, the area below the regional power lines that transect forests covers approximately 200 km^2^. The woody vegetation in these areas is cut every 5–10 years to prevent trees from encroaching on the power lines. If appropriately managed, these already disturbed areas may benefit pollinators, such as bees (Wojcik and Buchmann 2012) and butterflies (Berg et al. 2013), as they contain a higher floral diversity than the neighboring forests (Eldegard et al. 2015). Indeed, open‐canopy areas in forested landscapes increase the species richness and abundance of many bee species (Winfree et al. 2007; Hanula et al. 2015). However, while management strategies that increase the sun exposure in power line clearings may benefit thermophilic organisms, such as bees (Sydenham et al. 2014) and reptiles (Shine et al. 2002), organisms that require humid environments (e.g., gastropods and amphibians) may prefer more shaded habitats in late successional stages of power line clearings. If the aim of management is to improve habitat conditions for a wide variety of organisms, and thus maximize the positive effects on diversity, managers may therefore need to apply a combination of different management strategies to accommodate the habitat requirements of each organism group.

Even among bee species, habitat requirements differ substantially. Evaluating the success of management plans based solely on the effect on bee species richness and abundance is therefore not recommended as these indices may provide limited ecological information. Instead, these indices should be accompanied by measures of the functional diversity within species assemblages (Cadotte et al. 2011) as the functional and species diversity indices may reveal contrasting patterns to habitat conditions (Forrest et al. 2015). Indeed, the response of bee species to land use changes can be explained by functional traits, such as nest‐site locations, body size, floral specialization (Williams et al. 2010), and phenology (Sydenham et al. 2014), which, together with the bee phylogeny, provide important information about the distribution of bees along environmental gradients (Hoiss et al. 2012; Sydenham et al. 2015). Thus, an ideal outcome of habitat management aimed at promoting the diversity of bees should be an increased species richness and abundance, accompanied by an increased (or at least an unchanged) phylogenetic and functional diversity. While these outcomes should manifest themselves at the population level, the initial response to altered habitat conditions occurs at the behavioral level of individuals within the community (Wong and Candolin 2015). Differences in the number of species and individuals among treatments likely reflect habitat use by bees from the local species pool. Given that the individuals are free to choose habitat, a higher use of one habitat over another likely reflects that the more used habitat is more preferred and thus profitable. A contrasting attraction or avoidance of species to differently managed habitat patches within the spatial scale of the community can therefore reveal which of the management practices best accommodate the preferred habitat of bees from the local species pool.

We established a large‐scale field experiment to test how the species, phylogenetic, and functional diversity of wild bees responded to different management practices in power line clearings in a varying environmental context. At each study site (*n* = 19), we established three plots and randomly assigned either of three treatments to each plot: (1) Cut: all trees cut and left to decay in the clearing; (2) Cut + Remove: all trees cut and removed from the plot; and (3) Uncut: uncleared. The sites were distributed across a large geographic area covering an elevation gradient and a gradient in floral diversity. This allowed us to study how the environmental context affected the local species pool and thereby whether the outcome of management practices depended on the environmental context.

We sampled the bee community within each treatment plot in each site and hypothesized that:

Cleared plots (i.e., treatments Cut and Cut + Remove) should attract more bee species from the local species pool than uncleared plots (i.e., Uncut) as sun exposure is an important determinant of local bee species richness and abundance in boreal forest ecosystems (Sydenham et al. 2014). We expected to find the highest species richness of bees in plots where the woody debris was removed (Cut + Remove) and, consequently, a larger area of potential nesting sites exposed, as many bee species nest in sun‐exposed soils. Moreover, as local bee diversity may be positively related to floral diversity (Potts et al. 2003), we expected that the relative difference in the number of bee species and individuals between cleared and uncleared treatment plots would increase with local floral diversity. In contrast, as bee diversity normally decreases with elevation (Hoiss et al. 2012; Sydenham et al. 2015), we expected that the effect of clearing would decrease with elevation.

As habitat improvement increases the presence of habitat specialists in hedgerows (Kremen and M'Gonigle 2015), we also expected an increase in the number of species and individuals of bees after clearing plots with a high floral diversity to be accompanied by an increased phylogenetic and functional diversity. In contrast, we expected the phylogenetic and functional diversity to decrease with elevation because of reduced bee diversity with increasing elevation (Hoiss et al. 2012; Sydenham et al. 2015).

## Methods

### Study design and data collection

We identified 19 sites within the main power line grid in southeast Norway, which had a stretch of at least 200 m with substantial regrowth of trees below the power lines. Sites were located between latitudes 59.33°–61.12°N and longitudes 08.95–11.36°E at 48–536 m.a.s.l. The clearings varied in width from c. 40 to c. 80 m. All the sites had been subjected to the same management regime with manual clear‐cutting of all woody vegetation every 5–10 years, over large stretches of the corridor, where felled trees were left on site.

**Figure 1:**
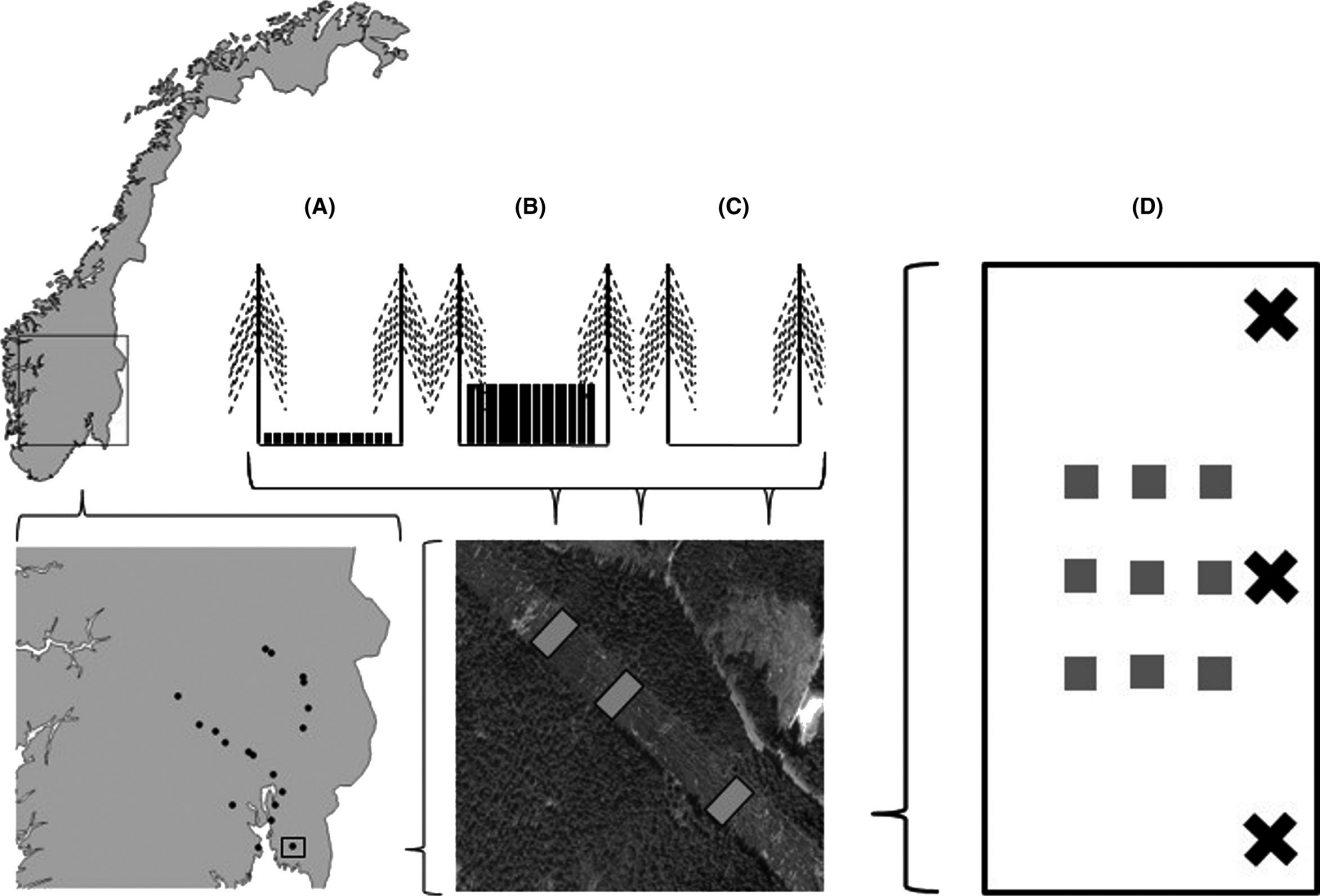
The geographic distribution of the 19 study sites located within power line clearings showing the three treatment plots (illustrated as cross‐sections of the power line clearing); (A) Cut: all trees cut and left to decay in the clearing, (B) Uncut: uncleared control and (C) Cut + Remove: all trees cut and removed from the plot, thereby exposing the ground to direct sunlight. We sampled bees with three flight interception traps (black crosses) in each treatment plot (D), and plant data in the nine 1‐m^2^ subplots within each of the three treatment plots (gray boxes).

At each site, three rectangular plots extending 30 m along the power line clearing and covering the whole width of the clearing were established at least 20 m apart. During the late autumn 2012 (16 sites) and early spring 2013 (3 sites), we randomly allocated one of the plots to three treatments: (1) Cut: all trees were cut and left to decay in the plot; (2) Cut + Remove: all trees were cut and immediately removed from the plot, that is, mimicking harvesting of biomass for, for example, biofuels; and (3) Uncut: the plot was left uncleared (Fig. 1).

In the center of each treatment plot, we placed nine 1‐m^2^ subplots 5 m apart in a regularly spaced grid pattern. We visually estimated the total cover of all forb species in the nine subplots within each of the three treatment plots at every site. We also estimated the cover of four Ericaceae shrub species (*Calluna vulgaris, Vaccinium myrtillus, V. vitis*‐*idaea,* and *V. uliginosum*), which are visited by the regionally common pollen specialists *Andrena fuscipes, A. lapponica,* and *Colletes succinctus*. We focused on forbs and ericaceous shrubs because the relative dominance of these groups may be an important determinant of bee species composition in forested ecosystems (Hanula et al. 2015; Sydenham et al. 2015). We calculated the species richness of forbs and ericaceous shrubs within each treatment plot by pooling all species occurring in the nine 1‐m^2^ subplots. The abundance of forbs and ericaceous shrubs was quantified by calculating their average cover per subplot in each treatment plot. The species richness of forbs was strongly positively correlated with the cover of forbs (*ρ* = 0.76) and negatively correlated with both the cover (*ρ* = −0.60) and species richness (*ρ* = −0.59) of Ericaceous shrubs. Thus, the gradient in forb species richness represented a gradient in floral diversity (from structurally simple, unproductive, Ericaceous shrub dominated; to structurally complex, productive, forb dominated), and we therefore only used species richness of forbs within treatment plots (mean = 10.56, min = 0, Q_25_ = 5, Q_50_ = 7, Q_75_ = 16, max = 30) to represent floral resource abundance and diversity.

Bees were sampled in 2013 throughout the entire flowering season, from snowmelt to the end of the foraging activity‐season in September. We installed three flight interception traps within each treatment plot. All traps were placed along the northernmost end of the treatment plot to maximize sun exposure. The flight interception traps consisted of two transparent Plexiglas screens (370 mm × 210 mm) that formed a cross, with a funnel and collecting bottle attached to it. The collecting bottle was filled with a 50:50 mixture of green propylene glycol and a drop of detergent. Every month we collected the bottles and immediately replaced them with new ones. This procedure ensured a continuous, standardized sampling among all sites throughout the entire season. We pooled all specimens sampled within a treatment plot and used the treatment plots as our sampling unit in the statistical analyses.

### Phylogenetic diversity indices

We constructed an ultrametric, polytomous, proxy of a phylogenic tree for the bee species collected in this study (Fig. S1) by clustering taxa according to published phylogenies (Danforth et al. 2013; Schmidt et al. 2015). Branch lengths were calculated following Grafen (1989) with the p‐parameter set to 0.25 in order to place the divergence of bee families early in the phylogeny as these evolved early in the phylogenetic history of bees (Cardinal and Danforth 2013).

We calculated the four indices (Table 1) proposed by Helmus et al. (2007) as measures of the phylogenetic diversity of bees occurring within the treatment plots. The phylogenetic species variability (PSV) quantifies the evolutionary distinctiveness of the species in a sample. The PSV equals one when all species in the community are equally unrelated to one another (i.e., the phylogeny is star shaped) and approaches zero as the species become increasingly related. The PSV is calculated as one minus the mean similarity in phylogenetic history (i.e., the mean off‐diagonal values in a phylogenetic variance–covariance matrix). The phylogenetic species richness (PSR) is the phylogenetically corrected species richness. It is calculated as the species richness multiplied by the PSV. The phylogenetic species evenness (PSE) is the abundance‐based PSV and is a measure of how evenly spread the individuals in a sample are, in terms of their evolutionary history. If all species have the same abundance, PSE equals PSV. Finally, the phylogenetic species clustering (PSC) is the PSV modified to provide information about how species in an assembly are clustered toward the tip of the phylogeny. The PSC approaches zero when the evolutionary distinctiveness of the nearest relatives in the community increases. A strength of the PSV‐based indices is that they are standardized against a star‐shaped phylogeny and therefore are not dependent on the regional species pool (Helmus et al. 2007). Prior to analyses, we removed all cleptoparasitic species from the data set as these species are highly host‐specific and therefore co‐occur with their hosts, although many of them (as in the case of the genus *Nomada*) are distantly related to their hosts. Including these species in the analyses could therefore obscure the effects of habitat conditions on the phylogenetic diversity of bee species assemblages. A total of 11 treatment plots were removed as they then contained fewer than two species, making it impossible to calculate the phylogenetic diversity indices. We used the picante library (Kembel et al. 2010) in R (R development core team 2014) to calculate the phylogenetic diversity indices (Table 1).

**Table 1:** Abbreviations used for phylogenetic (Helmus et al. 2007) and functional (Villèger et al. 2008) diversity indices used in this paper. See text for details

	Abbreviation
Phylogenetic diversity^a^	
Phylogenetic species variability	PSV
Phylogenetic species richness	PSR
Phylogenetic species evenness	PSE
Phylogenetic species clustering	PSC
Functional diversity^b^	
Functionally singular species richness	FSSR
Functional dispersion	FDis
Functional evenness	FEve
Community‐weighted mean	CWM
Proportion of oligolectics	None used
Proportion of aboveground nesters	None used
Intertegular distance (body size)	CWM ITD
Month of emergence	CWM emergence

a Cleptoparasites were excluded from analyses with phylogenetic diversity indices as response variables.

b The FDis, FEve, and CWM indices were abundance‐weighted as we were interested in the preference of individuals for different treatment plots. Cleptoparasites were excluded in the analyses on the proportions of oligoletics and aboveground nesters.

### Functional diversity indices

To explore whether treatments differed with respect to functional diversity, we tested whether the bees trapped in the different treatment plots constituted only a subset of the functional groups from the local (site) species assemblage. The functional dissimilarity among bee species was quantified based on five life‐history traits related to four main categories; foraging behavior, nesting behavior, month of emergence, as a measure of phenology, and the intertegular distance (ITD) as a measure of body size (Table S2). These traits were chosen as they are related to the responses of bees to disturbances (Williams et al. 2010; Sydenham et al. 2014; Forrest et al. 2015) and the ITD as it determines their foraging range (Greenleaf et al. 2007). We calculated four indices of functional diversity (i.e., FSSR, FDis, FEve, and CWM); the number of functionally distinct species (hereafter; functionally singular species richness; FSSR), which is the functional equivalent of species richness. To quantify the variation in trait values within treatment plots, we followed the approach of Forrest et al. (2015) and calculated the functional dispersion (FDis) of trait values. The functional evenness (FEve) was calculated to test how the skewness of trait values differed within and among sites (Villèger et al. 2008). Non‐Euclidean distances, due to the inclusion of categorical traits, were corrected using the Cailliez correction (Forrest et al. 2015). We also calculated the community‐weighted mean (CWM) trait value to test whether the attraction of bees to cleared sites depended on pollen specialization (i.e., polylectics vs. oligolectics), nesting behavior (i.e., above vs. belowground nesters), month of emergence, and body size (i.e., ITD). All the trait diversity indices were calculated using the FD library (Laliberte and Legendre 2010) in R. The indices were abundance‐weighted as we were interested in the distribution of individuals, belonging to different functional groups, among treatment plots.

### Statistical analyses

We analyzed the effects of the treatments on the response variables by fitting a generalized linear mixed effect models (GLMM) using the R library lme4 (Bates et al. 2015). For each response variable, we first fitted a full model which included the treatment‐specific interaction terms treatment × forb species richness, treatment × elevation, and the site‐specific interaction term forb species richness × elevation as well as the main effect terms of each variable and site identity as a random effect. The number of successful trap‐months (hereafter; sampling intensity) within each treatment plot was included as an offset variable to account for a difference in sampling intensity caused by destroyed traps (27 trap‐months missing of 684). We first used chi‐square tests in R to test whether including the sampling intensity significantly reduced the model deviance. If not, we refitted the model without sampling intensity as an offset variable. We then used likelihood ratio tests (LRTs) to conduct a sequential backward elimination of nonsignificant terms (*P* > 0.05, LRT statistics for all variables dropped from models as well as outputs from final models are listed in Tables S3, S5 and S6).

Analyses with either species richness or abundance of solitary bees as the dependent variable were run using GLMMs with Poisson‐distributed errors and log‐link functions. We analyzed the data both with and without cleptoparastic species (*i.e., Coelioxys, Nomada,* and *Sphecodes* spp.). Analyses with the PSR as the dependent variable were fitted using GLMMs with a Gamma distribution and a log‐link function, whereas the PSV, PSE, and PSC were fitted using linear mixed effect models.

The analyses with the FSSR (Table 1) as the response variable were run using negative binomial GLMMs whereas linear mixed effect models were fitted to the FDis, CWM ITD, and the CWM emergence. The proportion of belowground nesting bees and oligolectics (i.e., pollen specialists) were fitted using GLMMs with binomial distributions and logit‐link functions. Cleptoparasitic species were excluded from the single trait analyses on nesting behavior and lecty status as these species only indirectly depend on the resources sought by their hosts. We did not include sampling intensity as an offset variable in the analyses on proportions data, as these variables were already standardized by the abundance of bees within treatment plots and therefore readily comparable among treatments and sites. We tested for nonindependence among the main functional trait categories (ITD, lecty status, month of emergence, and nest location) possessed by each species (*n* = 63 species) using Spearman's rank correlation tests in the Hmisc (Harrell et al. 2015) library in R. This was carried out since the interpretation of analyses on individuals traits might be confounded by co‐variation among traits.

## Results

### Influence of experimental treatments and habitat conditions on species richness and abundance

**Figure 2:**
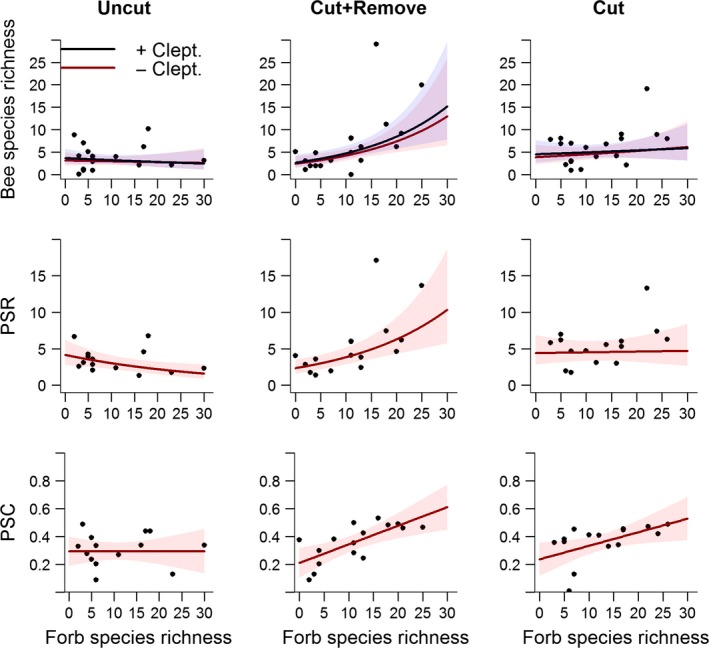
The effects of treatments (Uncut, Cut + Remove, and Cut) on the relationship between species richness, phylogenetic species richness (PSR), and phylogenetic species clustering (PSC) and forb species richness. The results were similar when cleptoparasites were excluded (‐ Clept.). Plots show fitted lines and 95% confidence intervals for GLMMs on the interaction term treatment × forb species richness (see text and Tables 2 and 3 for details).

We collected a total of 617 individuals and 63 species of solitary bees. The species richness (incl. cleptoparasites) differed among treatment plots, but the magnitude depended on the forb species richness within plots. This shows that the effect of removing the debris after clearings was strongest in the most productive (i.e., floristically diverse) sites (Fig. 2, Tables 2 and S3). Results for bee species richness were similar when cleptoparasites were removed (Fig. 2, Table 2) and also for the abundance of bees, regardless of the inclusion/exclusion of cleptoparasites (Table 2). There was also a decrease of species richness with elevation, but this did not differ among treatments (Fig. S4). See Table S3 for likelihood ratio tests statistics for dropped variables and the full GLMM outputs for the final models.

**Table 2:** Likelihood ratio tests for final models on bee species richness and abundance. Models were fitted using Poisson GLMMs with the number of successful trap‐months as an offset variable and site identity as a random effect. See Table S3 for LRT test statistics from the backward elimination of variables and model outputs from the final model

Response	Explanatory terms	LRT	df	*P*
Species richness (incl. Cleptoparasites)	Elevation	7.55	1	0.006
	Treatment × Forb species richness	13.26	2	0.001
Species richness (excl. Cleptoparasites)	Elevation	7.10	1	0.008
	Treatment × Forb species richness	9.00	2	0.011
Bee abundance (incl. Cleptoparasites)	Elevation	4.88	1	0.027
	Treatment × Forb species richness	33.39	2	<0.001
Bee abundance (excl. Cleptoparasites)	Elevation	4.70	1	0.030
	Treatment × Forb species richness	27.20	2	<0.001

### Influence of experimental treatments and habitat conditions on phylogenetic diversity

The phylogenetic species richness (PSR) differed among treatments within sites, but the differences depended on the forb species richness within treatment plots and elevation (Fig. 2, Table 3). Inclusion of the interaction term treatment type × elevation (Table S5) significantly improved the model fit (*χ* ^2^ = 8.44, *P* < 0.038) and was marginally significant (df = 2, LRT = 5.864, *P* = 0.053).

**Table 3:** Likelihood ratio tests for final models on the phylogenetic diversity within treatment plots in power line clearings. The phylogenetic species richness (PSR) was fitted using Gamma GLMMs with site identity as a random effect. The phylogenetic species clustering (PSC) was fitted using a linear mixed effect model. See Table S5 for LRT test statistics from the backward elimination of variables and model outputs from the final model

Response	Explanatory terms	LRT	df	*P*
Phylogenetic species richness (PSR)	Treatment × Forb species richness	24.40	2	<0.001
	Treatment × Elevation	5.86	2	0.053
Phylogenetic species clustering (PSC)	Treatment × Forb species richness	7.61	2	0.022
	Forb species richness × Elevation	4.34	1	0.037

The increased PSR was not paralleled by a selective bias against specific taxonomic groups as neither the phylogenetic species variability (PSV) or evenness (PSE) varied systematically along the gradient in forb species richness, elevation, or among treatments (Table S5). In contrast, the phylogenetic species clustering (PSC) differed among treatments and increased with forb species richness in the Cut+Remove and Cut treatments but not in the Uncut treatment (Fig. 2, Table 3). Moreover, the significant interaction between forb species richness and elevation showed that the PSC increased with forb species richness and that the slope of this relationship increased with elevation (Table 3, Fig. S4). See Table S5 for likelihood ratio test statistics from the backward elimination of variables and full model outputs from the final models for PSR and PSC.

### Influence of experimental treatments and habitat conditions on functional diversity

**Figure 3:**
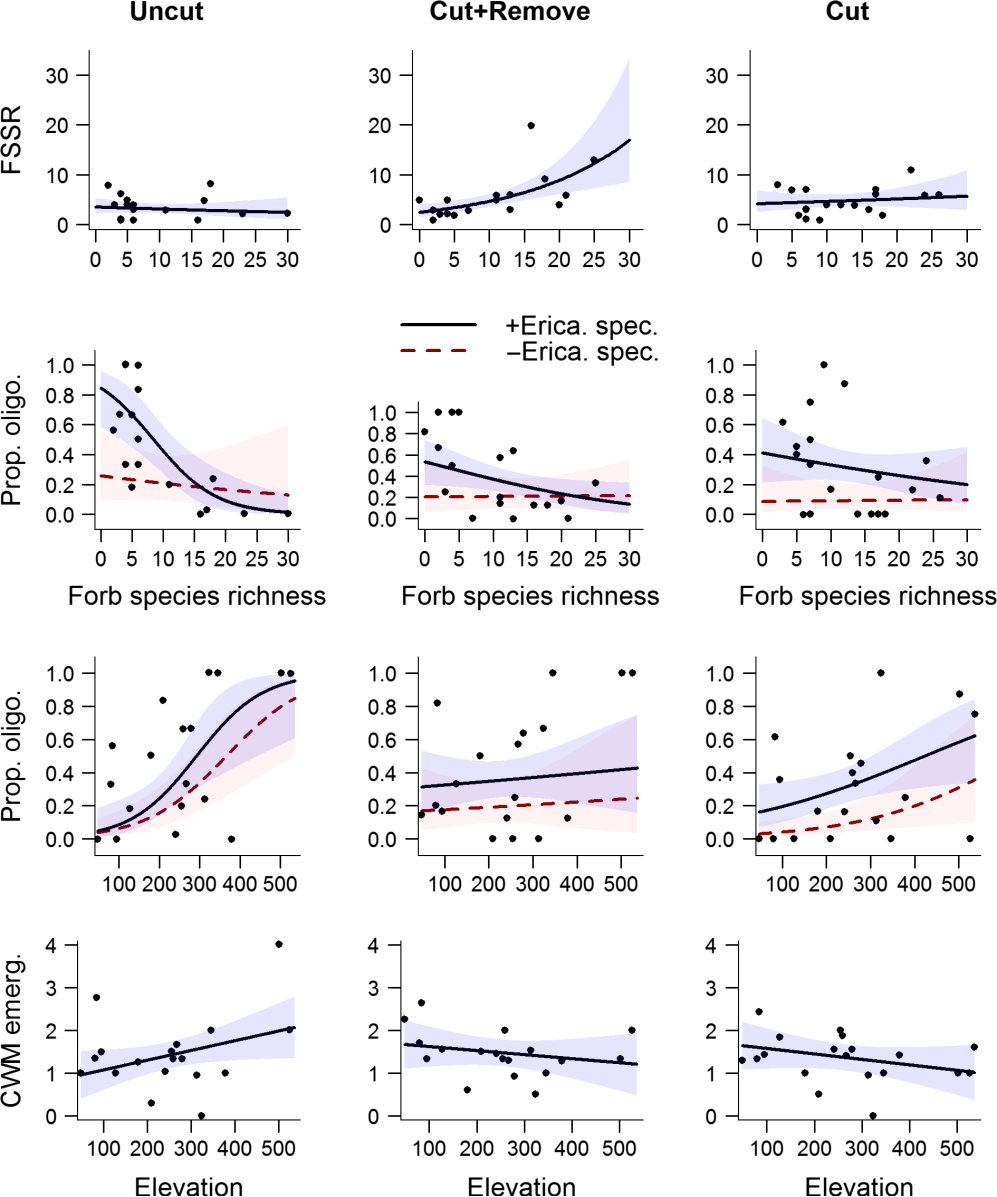
The effects of treatments (Uncut, Cut + Remove, and Cut) on the relationship between the functionally singular species richness (FSSR), proportion of oligolectics (prop. oligo.), and community‐weighted mean month of emergence (CWM emerg.) and environmental conditions (forb species richness and elevation). Relationships are also shown when Ericaceae specialists were excluded from the analyses on Prop. Olig. (‐ Erica. spec.). Plots show fitted lines and 95% confidence intervals for GLMMs.

The functionally singular species richness (FSSR) increased with forb species richness in the Cut + Remove treatment whereas this relationship did not occur in the Cut and Uncut treatments (Fig. 3, Table 4). There was also a positive interaction between forb species richness and elevation, suggesting that the importance of forb species richness for FSSR increased with elevation (Fig. S4, Table 4).

**Table 4:** Likelihood ratio tests for final models on the functional diversity within treatment plots in power line clearings. The functionally singular species richness (i.e., FSSR; the number of functionally unique species) was fitted using a negative binomial GLMM whereas the functional evenness (FEve) and community‐weighted mean body size of bee individuals (ITD) were fitted using linear mixed effect models. Analyses with the proportion of belowground nesting and oligolectic bee individuals were run using GLMMs with binomial distributions. See Table S6 for LRT test statistics from the backward elimination of variables and model outputs from the final model

Response	Explanatory terms	LRT	df	*P*
Functionally singular species richness (FSSR)	Treatment type × Forb species richness	12.50	2	0.002
	Forb species richness × Elevation	5.14	1	0.023
Functional Evenness (FEve)	Forb species richness	12.65	1	<0.001
	Elevation	3.97	1	0.046
Mean body size of bee individuals (ITD)	Forb species richness	9.46	1	0.002
	Elevation	8.88	1	0.003
Proportion belowground nesting bee individuals	Treatment type	17.72	2	<0.001
Proportion of Oligolectic bee individuals	Treatment type × Forb species Richness	10.34	2	0.006
	Treatment type × Elevation	9.06	2	0.011
Mean emergence time of individuals	Forb species richness	4.92	1	0.027
	Treatment type × Elevation	10.95	2	0.004

We found no systematic change in the FDis within or among sites (Table S6). However, the FEve decreased with forb species richness and increased with elevation so that the individuals sampled in treatment plots at high elevations with a low forb species richness had the most evenly distributed functional trait distributions (Fig. S4, Table 4). The CWM ITD of individuals increased with elevation and decreased with forb species richness (Fig. S4, Table 4). These responses were site‐specific as no interactions occurred between the environmental variables and treatment (Table S6).

**Figure 4:**
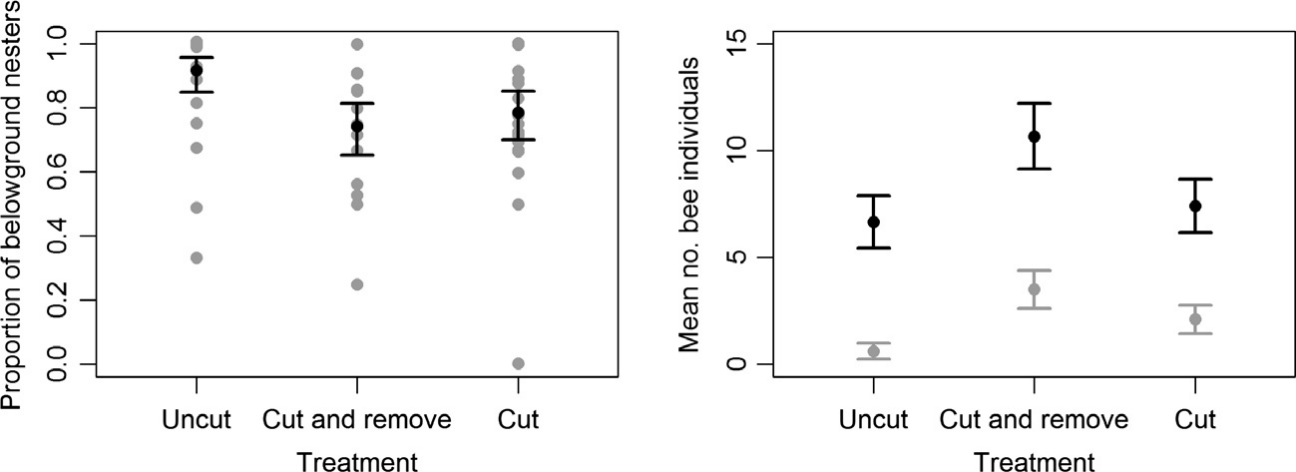
The proportion of belowground nesting bee individuals differed among treatment plots and was lowest in the two cleared treatment plots (Cut and Cut+Remove). Black dots and whiskers are predicted values and 95% CI limits. Gray dots are observed values. The right panel shows the mean number of belowground nesting (black) and aboveground nesting (gray) bee individuals per treatment type with 95% CI limits estimated using individual Poisson GLMs for each of the six nesting behavior–treatment combination. See Table 4 for test statistics.

The proportion of belowground nesting bee individuals was lower in the two cleared treatment types than in the Uncut treatment (Fig. 4, Table 4), but was not related to elevation or forb species richness (Table S6). The proportion of pollen specialists (oligolectics) decreased with forb species richness and increased with elevation and these relationships were most pronounced in the Uncut treatment type, indicating an effect of treatment. However, the relationships between the proportion of oligolectic bees and the interaction terms treatment × forb species richness (Fig. 3, df = 2, LRT = 0.36, *P* = 0.836) and treatment × elevation (df = 2, LRT = 5.15, *P* = 0.076) were not significant when Ericaceae specialists were removed from the analysis. The CWM emergence increased with elevation in the Uncut treatment plots but decreased with elevation in the Cut + Remove and Cut treatment plots (Table 4, Fig. 3). The CWM emergence also decreased with forb species richness (Table 4, Fig. S4). See Table S6 for likelihood ratio test statistics from the backward elimination of variables and full model outputs from the final models fitted to the functional diversity indices.

## Discussion

The effect of habitat management on bee diversity was modified by the environmental context. The cleared treatment plots in power lines (treatments Cut and Cut + Remove) attracted more species and individuals than noncleared plots (Uncut) and this effect increased with forb species richness. Contrary to our expectations, the effect of treatments did not change with elevation, and the phylogenetic similarity among species increased following the clearing of power lines in plots with a high species richness of forbs. However, the increase in phylogenetic similarity was driven by a parallel packing of species within several taxonomic groups. This was also supported in that the Cut and Cut + Remove treatments were slightly less dominated by belowground nesting bees than the Uncut treatment and that the proportion of oligolectic individuals was more stable along the forb species richness and elevation gradients in the two cleared treatments, compared to the Uncut treatment. There was a significant correlation among several of the trait categories (Table 5). Pollen specialists and aboveground nesters generally emerged later in the season, and pollen generalists tended to be smaller than pollen specialists. These interdependencies meant that the single trait analyses have to be interpreted in concert as some trait–environment relationships might have been confounded by other traits than the one being tested.

**Table 5:** Spearman's rank correlations among the main functional trait categories. *P*‐values are given in parenthesis. The body size of species (intertegular distance; ITD) and the month of emergence (Emergence) are numerical values. The Lecty status and Nest location are binary given the value 1 for polylectics and belowground nesters, respectively

	Emergence	ITD	Lecty status
ITD	0.05 (0.70)	1	−0.33 (0.01)
Lecty status	−0.30 (0.02)	−0.33 (0.01)	1
Nest location	−0.35 (<0.01)	−0.03 (0.79)	−0.16 (0.22)

### Influence of management practices on species richness and abundance of bees

The cleared treatment plots attracted more bee species and individuals from the local species pools than the noncleared plots, and the effect of clearing and debris removal was greatest in sites with a high species richness of forbs. In contrast, in the ericaceous shrub dominated (i.e., low forb species richness) sites, the treatment effect was negligible (Fig. 2, Table 2). This may indicate that bee species in our study preferred different successional stages, as previously shown for bees along temporal gradients in fire history (Moretti et al. 2009; Ricotta and Moretti 2011). In our system, this preference could be related to floral specialization in that Ericaceae‐affiliated species are less affected by clearing regrowth than forb‐affiliated species. For instance, the common Ericaceae specialist *Andrena lapponica* is frequently foraging on bilberry (*Vaccinium myrtillus*) in shaded *Picea abies* forests (pers. obs.), whereas the majority of solitary bees prefer sun‐exposed areas in this ecosystem (Sydenham et al. 2014) with a diverse flora (Hanula et al. 2015; Sydenham et al. 2015). This suggests that forb‐dominated power line clearings have a greater potential for boosting local bee diversity than those dominated by ericaceous shrubs.

In addition to the influence of clearing and floral diversity, elevation was negatively related to the species richness and abundance of bees within treatment plots (Table 2, Table S3, Fig. S4). The species and functional diversity of wild bee species assemblages have previously been shown to decrease with elevation (Hoiss et al. 2012; Sydenham et al. 2015). In contrast to our expectations, the influence of elevation on bee species richness did not differ among the three experimental treatments (Fig. S4, Table 2). Scheper et al. (2013) found that the effect of habitat management in landscapes with little or no semi‐natural habitats was negligible due to low species densities. The same may have occurred in the power line clearings at high elevations, where a low species richness in sites could mask potential differences in habitat quality among treatments.

### Influence of management practices on the phylogenetic composition

The attraction of bee species to cleared plots with a high forb species richness also led to an increased phylogenetic species richness (PSR, Fig. 2), as would be expected as this index was correlated to species richness in our data set. We expected the phylogenetic species variability (PSV) and evenness (PSE) to change with forb species richness, treatment, or their interaction, as long‐tongued bees prefer later successional habitats than distantly related short‐tongued bees (Moretti et al. 2009; Ricotta and Moretti 2011), but we found no such relationships. That the phylogenetic species clustering (PSC) increased with forb species richness in both clearing treatments, but not in the Uncut treatment (Table 3, Fig. 2), suggests that taxa did not discriminate between the clearing treatments and that most clades were present in areas that had plots with a high forb diversity. The preference for cleared treatment plots could be caused by the increased solar radiation in such plots, as sun exposure is an important determinant of solitary bee species richness in field margins along forest edges (Sydenham et al. 2014). In addition to increased local temperature, the direct sunlight could also increase the number of species flowering, and their flower density, in cleared plots, thereby increasing the diversity of foraging resources. As different taxa of bees tend to show distinct floral preferences (Potts et al. 2003), an increased diversity of floral resources could attract more species, from distantly related taxa, to the same treatment plot.

### Influence of management practices on the functional diversity

The effects of treatments on the functional diversity of bees depended on elevation and on the forb species richness within plots. The response of the functional diversity of bees to habitat conditions may contrast that of species diversity if the habitat selects for bees with certain traits (Forrest et al. 2015). Indeed, while the response of the FSSR to treatments mirrored that of the species richness and PSR (Figs. 2 and 3, Tables 2–4) there were also trait‐specific responses (Figs. 3 and 4, Table 4). We found a lower proportion of belowground nesting bees in the cleared plots despite these plots contained a larger area of sun exposed soil, which is attractive to nest seeking females (Potts et al. 2005). The preference for cleared plots may therefore have been driven mainly by foraging resources as floral diversity is an important driver of bee diversity (Potts et al. 2003). Moreover, differences among treatments in the relationships between the proportion of oligolectic bees and elevation and forb species richness were only significant when Ericaceae specialists were included in the analyses (Fig. 3). This suggests that Uncut treatment plots with a low diversity of foraging resources and at high elevations tended to be more dominated by Ericaceae specialists than the cleared plots. Oligolectics tended to emerge later in the season than polylectics (Table 5). That the CWM emergence increased with elevation in the Uncut treatment and decreased in two cleared treatment types (Fig. 3) may therefore have been caused by early emerging, polylectic, bees preferring the cleared plots at high elevations. At high elevations, this preference could be due to an earlier onset of flowering, resulting from increased sun exposure to the ground.

In addition to the effects of treatment, the functional diversity was also influenced by environmental conditions irrespective of treatments (Fig. S4). As the species richness decreased with elevation (Fig. S4), it surprised us that the FSSR increased with the interaction between elevation and forb species richness (Fig. S4). However, the decrease in functional evenness (FEve), with forb species richness and increase with elevation suggests an even trait distribution at high elevation sites with a high forb species richness. This could lead to an increased FSSR in treatment plots at high elevations areas with a diverse forb community. That the CWM ITD increases with elevation (Table 4, Fig. S4) was also found by Hoiss et al. (2012), who suggested that the relationship could be caused by large species being better at thermoregulating and able to fly under poorer weather conditions or that large species can forage over greater distances (Greenleaf et al. 2007). A decrease in CWM ITD with forb species richness was also found in another study in power line clearings in Norway (Sydenham et al. 2015), and was likely caused by sites with a high dominance of Ericaceous shrubs being species poor and dominated by the relatively large Ericaceae specialists (mean ITD = 2.67 ± 0.47 mm vs. 2.1 ± 0.75 mm). This could also explain why the mean month of emergence decreased with forb species richness (Fig. S4) as sites with a high species richness of forbs would be more likely to provide foraging resources for earlier emerging polylectics.

### Implications for habitat management

Different types of habitats host‐specific bee species assemblages (Murray et al. 2012). Thus, it should be of high conservation priority to develop habitat‐type specific management strategies in potentially bee friendly habitats, such as power line clearings (Russell et al. 2005). In the European Union (EU), the implementations of such management plans in agricultural landscapes are motivated through the EU Agri Environmental Schemes. These include establishing flower‐strips along field margins and restoring hedgerows, which increases local bee diversity (Kremen and M'Gonigle 2015). In contrast, no environmental schemes have been proposed for power line clearings that transect forests, although they cover vast areas and host diverse plant (Wagner et al. 2014; Eldegard et al. 2015), bee (Russell et al. 2005), and butterfly (Berg et al. 2013) species assemblages. However, the impact of management practices depends on the environmental context and is greatest in landscapes of intermediate complexity that contain source habitats from which species can recolonize restored areas (Scheper et al. 2013). Our findings are in agreement with Scheper et al. (2013) in that the largest increase in diversity occurs in sites with a high species richness and they highlight the importance of testing management schemes under different environmental conditions prior to establishment.

Our findings suggest that changes in management practices, that is, removing debris after clearing, create a preferred habitat for bees and whether these translate into an increased pollen provisioning for offspring may enhance bee diversity in power line clearings. However, it should be noted that this study was limited to the diversity of solitary bees, and the suggested management advice might have different effects on other organisms. In order to mitigate negative effects on, for example, the local diversity of decomposers, the woody debris could be left on site, but collected in heaps, thereby creating a heterogeneous environment that benefits both bees and organisms that depend on dead wood. Moreover, the responses measured in this study are likely to be a mixture of population‐level responses occurring at the site level, and behavioral‐level responses occurring within sites. Future studies of bees should aim to test whether behavioral‐level responses, such as those documented in this study, manifest themselves at the population level.

One of our treatments mimicked an labor‐intensive management practice with removal of woody debris from the area after management clearing. Unless the debris is harvested and sold for biofuels or other products, removing the woody debris will increase the net cost of clearing the power line clearings. The most cost‐effective solution would be to allocate this treatment to sites where the benefit is expected to have the greatest positive effect on bee diversity. Our results show that the largest effect is gained in areas with a relatively high forb diversity. In boreal forest landscapes, these are typically areas with a high site productivity and low soil moisture. Moreover, as the decrease in species richness and abundance with elevation was not affected by management practice, managers should remove debris primarily at low elevations as these areas have the highest bee diversity. Logistically, this will likely reduce the cost of intensified management as low elevational sites are generally more easily accessible.

## Data Accessibility

Data available from the Dryad Digital Repository: http://dx.doi.org/10.5061/dryad.qj4h3

## Conflict of Interest

None declared.

## Supporting information


**Figure S1.** The hypothesized phylogenetic tree used in this study.

**Table S2.** List of functional traits used in this study and their assignment to species.

**Table S3.** Backward elimination of variables and final model outputs from analyses of species richness and abundance.

**Figure S4.** Influences of environmental conditions on the bee diversity in treatment plots.

**Table S5.** Backward elimination of variables and final model outputs from analyses of phylogenetic diversity.

**Table S6.** Backward elimination of variables and final model outputs from analyses of functional diversity.
