# Supplementary figures and images for: The effects of habitat management on the species, phylogenetic and functional diversity of bees are modified by the environmental context
Source: Ecol Evol. 2016 Jan 18;6(4):961–73. doi: 10.1002/ece3.1963 (PMC4761776; doi:10.1002/ece3.1963)

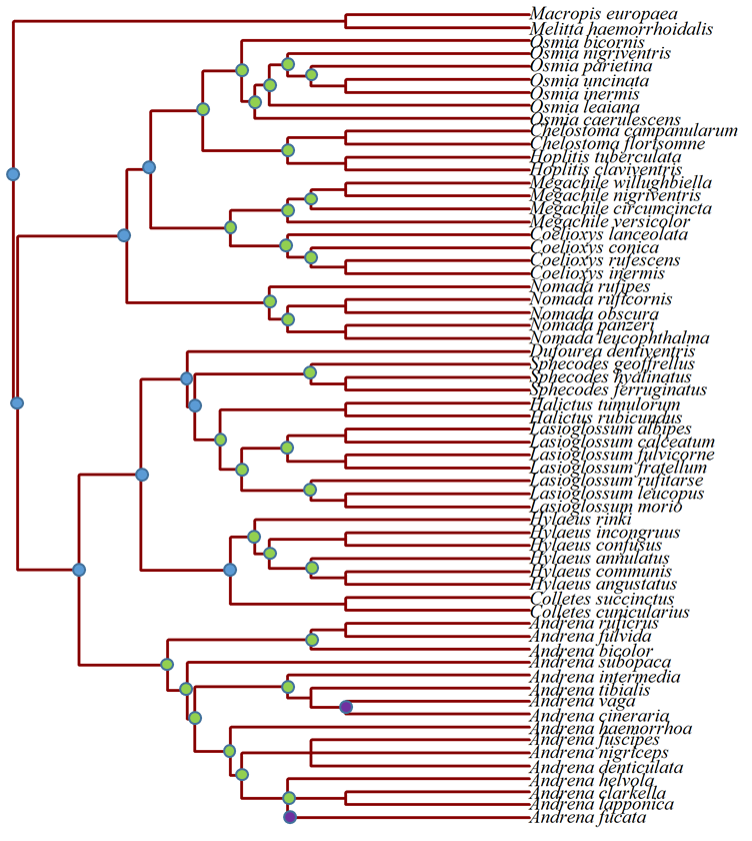

Supplement: Supplementary file 1 — Figure S1. The hypothesized phylogenetic tree used in this study. [file ECE3-6-0961-s001.tif]

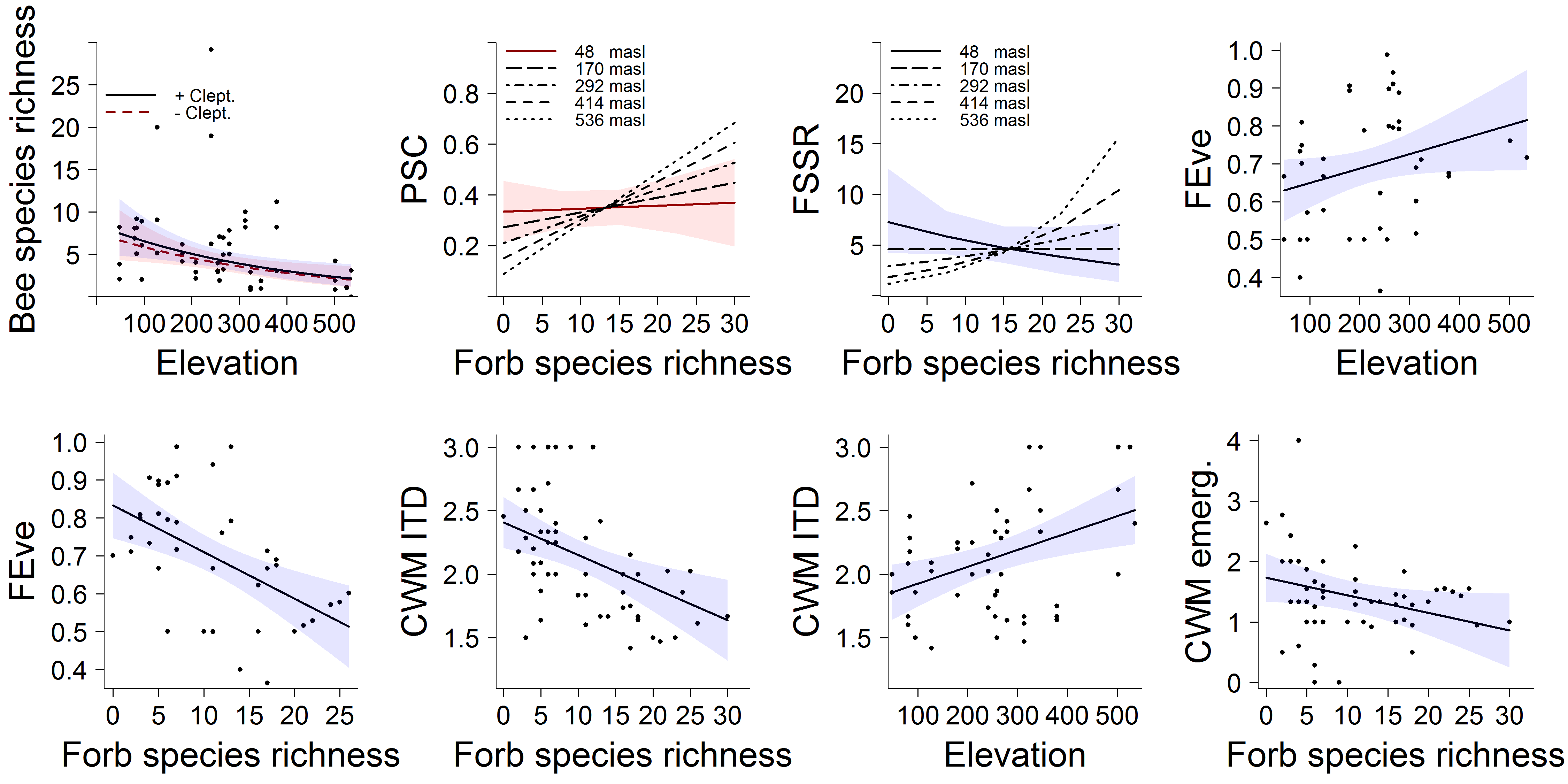

Supplement: Supplementary file 4 — Figure S4. Influences of environmental conditions on the bee diversity in treatment plots. [file ECE3-6-0961-s004.bmp]
