# Supplementary material for: The effects of habitat management on the species, phylogenetic and functional diversity of bees are modified by the environmental context
Source: Ecol Evol. 2016 Jan 18;6(4):961–73. doi: 10.1002/ece3.1963 (PMC4761776; doi:10.1002/ece3.1963)
Supplement: Supplementary file 2 — Table S2. List of functional traits used in this study and their assignment to species. [file ECE3-6-0961-s002.docx]

Table S2.1. Summary table of the functional traits included in this study. Cleptoparasites were assigned the exact same trait values as their hosts since they indirectly depend on these.

| Trait category | | | | | Variable type | Trait range |
| --- | --- | --- | --- | --- | --- | --- |
|  | Foraging behaviour | | | |  |  |
|  |  | Pollen specialist | | | Factor | Yes vs. No |
|  |  | Host plants for specialists | | | | |
|  |  |  |  | Asteraceae | Factor | Yes vs. No |
|  |  |  |  | Campanulaceae | Factor | Yes vs. No |
|  |  |  |  | Fabaceae | Factor | Yes vs. No |
|  |  |  |  | *Lysimachia* | Factor | Yes vs. No |
|  |  |  |  | *Raununculus* | Factor | Yes vs. No |
|  |  |  |  | *Salix* | Factor | Yes vs. No |
|  |  |  |  | Ericaceae | Factor | Yes vs. No |
|  | Nesting behaviour | | | | | |
|  |  | Above vs. below ground | | | Factor | Yes vs. No |
|  |  | Clepto-parasitic | | | Factor | Yes vs. No |
|  |  |  | Host genus for clepto-parasites | | Factor | *Andrena, Anthophora, Lasioglossum, Megachile* |
|  | Phenology | | | | | |
|  |  | Emergence | | | Numeric | March = 0, April = 1, May = 2, June =3, July = 4, August = 5. |
|  | Body size | | | | Numeric | Mean = 2.17, min = 1, max = 4 mm |

Table S2.2 The trait values assigned to each species. Species were designated as either polylectic (Poly = 1) or oligolectic (Poly = 0). For oligolectics we also specified the taxonomic affiliation of plants pollen is collected from. Species were also assigned a trait according to their nesting behaviour; i.e. below ground nesters (Nestbelow = 1) vs. above ground nesters (Nestbelow = 0). The month of emergence for species was included as a numerical variable (Table S2.1). Cleptoparasites were assigned an extra trait value according to the genera of their hosts (Clepto). We measured the intertegular distance (ITD) up to 10 female individuals of each species. We then used the mean ITD for each species, rounded to the nearest mm to account for measurement errors.

| Species | Poly | Oligo | Nestbelow | Emergence | Clepto | ITD |
| --- | --- | --- | --- | --- | --- | --- |
| *Andrena bicolor* | 1 | FALSE | 1 | April | FALSE | 2 |
| *A. cineraria* | 1 | FALSE | 1 | April | FALSE | 3 |
| *A. clarkella* | 0 | Salix | 1 | March | FALSE | 3 |
| *A. denticulata* | 0 | Asteraceae | 1 | July | FALSE | 2 |
| *A. fucata* | 1 | FALSE | 1 | April | FALSE | 2 |
| *A. fulvida* | 1 | FALSE | 1 | May | FALSE | 2 |
| *A. fuscipes* | 0 | Ericaceae | 1 | August | FALSE | 2 |
| *A. haemorrhoa* | 1 | FALSE | 1 | April | FALSE | 3 |
| *A. helvola* | 1 | FALSE | 1 | April | FALSE | 2 |
| *A. intermedia* | 0 | Fabaceae | 1 | June | FALSE | 2 |
| *A. lapponica* | 0 | Ericaceae | 1 | May | FALSE | 3 |
| *A. nigriceps* | 1 | FALSE | 1 | June | FALSE | 2 |
| *A. ruficrus* | 0 | Salix | 1 | March | FALSE | 2 |
| *A. subopaca* | 1 | FALSE | 1 | April | FALSE | 2 |
| *A. tibialis* | 1 | FALSE | 1 | April | FALSE | 3 |
| *A. vaga* |  | Salix | 1 | March | FALSE | 3 |
| *Chelostoma campanularum* | 0 | Campanulaceae | 0 | July | FALSE | 1 |
| *C. florisomne* | 0 | Raununculus | 0 | May | FALSE | 2 |
| *Coelioxys conica* | 1 | FALSE | 0 | May | Megachile | 3 |
| *C. inermis* | 1 | FALSE | 0 | May | Megachile | 2 |
| *C. lanceolata* | 1 | FALSE | 0 | June | Megachile | 3 |
| *C. rufescens* | 1 | FALSE | 1 | June | Anthophora | 3 |
| *Colletes cunicularius* | 0 | Salix | 1 | March | FALSE | 3 |
| *C. succinctus* | 0 | Ericaceae | 1 | August | FALSE | 3 |
| *Dufourea dentiventris* | 0 | Campanulaceae | 1 | July | FALSE | 2 |
| *Halictus rubicundus* | 1 | FALSE | 1 | April | FALSE | 2 |
| *H. tumulorum* | 1 | FALSE | 1 | April | FALSE | 1 |
| *Hoplitis claviventris* | 1 | FALSE | 0 | June | FALSE | 2 |
| *H. tuberculata* | 1 | FALSE | 0 | June | FALSE | 2 |
| *Hylaeus angustatus* | 1 | FALSE | 0 | June | FALSE | 1 |
| *H. annulatus* | 1 | FALSE | 0 | June | FALSE | 1 |
| *H. communis* | 1 | FALSE | 0 | May | FALSE | 1 |
| *H. confusus* | 1 | FALSE | 0 | May | FALSE | 1 |
| *H. incongruus* | 1 | FALSE | 0 | May | FALSE | 2 |
| *H. rinki* | 1 | FALSE | 0 | June | FALSE | 1 |
| *Lasioglossum albipes* | 1 | FALSE | 1 | April | FALSE | 2 |
| *L. calceatum* | 1 | FALSE | 1 | March | FALSE | 2 |
| *L. fratellum* | 1 | FALSE | 1 | April | FALSE | 1 |
| *L. fulvicorne* | 1 | FALSE | 1 | March | FALSE | 2 |
| *L. leucopus* | 1 | FALSE | 1 | April | FALSE | 1 |
| *L. morio* | 1 | FALSE | 1 | April | FALSE | 1 |
| *L. rufitarse* | 1 | FALSE | 1 | April | FALSE | 1 |
| *Macropiseuropaea* | 0 | Lysimachia | 1 | July | FALSE | 3 |
| *Megachile circumcincta* | 1 | FALSE | 0 | May | FALSE | 3 |
| *M. nigriventris* | 0 | Fabaceae | 0 | June | FALSE | 4 |
| *M. versicolor* | 1 | FALSE | 0 | May | FALSE | 3 |
| *M. willughbiella* | 1 | FALSE | 0 | June | FALSE | 3 |
| *Melitta haemorrhoidalis* | 0 | Campanulaceae | 1 | July | FALSE | 3 |
| *Nomada leucophthalma* | 1 | FALSE | 1 | March | Andrena | 2 |
| *N. obscura* | 1 | FALSE | 1 | March | Andrena | 2 |
| *N. panzeri* | 1 | FALSE | 1 | March | Andrena | 2 |
| *N. ruficornis* | 1 | FALSE | 1 | March | Andrena | 2 |
| *N. rufipes* | 1 | FALSE | 1 | July | Andrena | 2 |
| *Osmia bicornis* | 1 | FALSE | 0 | April | FALSE | 3 |
| *O. caerulescens* | 1 | FALSE | 0 | May | FALSE | 2 |
| *O. inermis* | 1 | FALSE | 0 | May | FALSE | 2 |
| *O. leaiana* | 0 | Asteraceae | 0 | June | FALSE | 3 |
| *O. nigriventris* | 1 | FALSE | 0 | April | FALSE | 3 |
| *O. parietina* | 1 | FALSE | 0 | May | FALSE | 2 |
| *O. uncinata* | 1 | FALSE | 0 | April | FALSE | 2 |
| *Sphecodes ferruginatus* | 1 | FALSE | 1 | May | Lasioglossum | 2 |
| *S. geoffrellus* | 1 | FALSE | 1 | May | Lasioglossum | 1 |
| *S. hyalinatus* | 1 | FALSE | 1 | April | Lasioglossum | 1 |
