# Supplementary material for: The effects of habitat management on the species, phylogenetic and functional diversity of bees are modified by the environmental context
Source: Ecol Evol. 2016 Jan 18;6(4):961–73. doi: 10.1002/ece3.1963 (PMC4761776; doi:10.1002/ece3.1963)
Supplement: Supplementary file 3 — Table S3. Backward elimination of variables and final model outputs from analyses of species richness and abundance. [file ECE3-6-0961-s003.docx]

Table S3.1 Backward elimination of variables from the full models. Response variables were: The species richness (incl. cleptoparasites), species richness (excl. cleptoparasites), bee abundance (incl. cleptoparasites) and bee abundance (excl. cleptoparasites) abbreviated as SR_incl_, SR_excl_, Ab_incl_ and Ab_excl._, respectively. The explanatory variables in the full models were: Treatment type, Forb species richness, Elevation, Treatment type × Forb species richness, Treatment type × Elevation, Forb species richness × Elevation and log(Number of Trap months) was included as an offset variable. In the first step (step 1) the full model was tested against a full model where the offset variable was excluded by comparing the deviance statistics between the models using χ^2^ tests. The offset variable was included if it decreased the deviance , and dropped if that increased the deviance. Variables with p-values > 0.05 were dropped from the model. Outputs from the final models are shown in table S3.2.

| Response | Explanatory and offset variables | LRT for variable | step |
| --- | --- | --- | --- |
| SR_incl._ | Offset variable: *included* | χ^2^ = 0.67, p < 0.001 | 1 |
|  | Treatment type × Elevation | df = 2, LRT = 0.20, p = 0.906 | 2 |
|  | Forb species richness × Elevation | df = 1, LRT = 3.08, p = 0.079 | 3 |
| SR_excl._ | Offset variable: *included* | χ^2^ = 0.35, p < 0.001 | 1 |
|  | Treatment type × Elevation | df = 2, LRT = 0.15, p = 0.928 | 2 |
|  | Forb species richness × Elevation | df = 1, LRT = 2.70, p = 0.101 | 3 |
| AB_incl._ | Offset variable: *included* | χ^2^ = 6.50, p < 0.001 | 1 |
|  | Treatment × Elevation | df = 2, LRT = 1.495, p = 0.473 | 2 |
|  | Forb species richness × Elevation | df = 1, LRT = 1.53, p = 0.217 | 3 |
| AB_excl._ | Offset variable: *included* | χ^2^ = 6.02, p < 0.001 | 1 |
|  | Treatment × Elevation | df = 2, LRT = 1.170, p = 0.557 | 2 |
|  | Forb species richness × Elevation | df = 1, LRT = 1.10, p = 0.293 | 3 |

Table S3.2 Final model outputs from Generalized Linear Mixed effect Models (GLMMs) on the species richness and abundance of solitary bees in power line clearings. Models were run with Poisson distributed errors and a log-link function.

| Bee species richness (*Including cleptoparasites)* | | | | β | SE | z | P |
| --- | --- | --- | --- | --- | --- | --- | --- |
|  |  | Intercept (Uncut) | | -0.455 | 0.333 | -1.37 | 0.172 |
|  |  | Cut-Remove | | -0.328 | 0.296 | -1.11 | 0.268 |
|  |  | Cut | | 0.206 | 0.29 | 0.71 | 0.477 |
|  |  | Forb Rich | | -0.014 | 0.018 | -0.76 | 0.446 |
|  |  | Elevation | | -0.003 | 0.001 | -2.83 | 0.005 |
|  |  | Cut-Remove × Forb Rich | | 0.072 | 0.022 | 3.35 | 0.001 |
|  |  | Cut × Forb Rich | | 0.023 | 0.021 | 1.09 | 0.274 |
|  |  | Random effects: | | σ | SD | Obs. | Sites |
|  |  |  | Site identity | 0.209 | 0.458 | 57 | 19 |
| Bee species richness (*Excluding cleptoparasites)* | | | | β | SE | z | P |
|  |  | Intercept (Uncut) | | -0.641 | 0.341 | -1.882 | 0.060 |
|  |  | Cut-Remove | | -0.262 | 0.305 | -0.858 | 0.391 |
|  |  | Cut | | 0.207 | 0.300 | 0.692 | 0.489 |
|  |  | Forb Rich | | -0.007 | 0.019 | -0.356 | 0.722 |
|  |  | Elevation | | -0.002 | 0.001 | -2.648 | 0.008 |
|  |  | Cut-Remove × Forb Rich | | 0.063 | 0.022 | 2.854 | 0.004 |
|  |  | Cut × Forb Rich | | 0.022 | 0.021 | 1.046 | 0.295 |
|  |  | Random effects: | | σ | SD | Obs. | Sites |
|  |  |  | Site identity | 0.197 | 0.444 | 57 | 19 |
| Bee abundance (*Including cleptoparasites)* | | | | β | SE | z | P |
|  |  | Intercept (Uncut) | | 0.198 | 0.421 | 0.471 | 0.638 |
|  |  | Cut-Remove | | -0.292 | 0.234 | -1.244 | 0.213 |
|  |  | Cut | | 0.338 | 0.231 | 1.466 | 0.143 |
|  |  | Forb Rich | | -0.025 | 0.014 | -1.763 | 0.078 |
|  |  | Elevation | | -0.003 | 0.001 | -2.294 | 0.022 |
|  |  | Cut-Remove × Forb Rich | | 0.076 | 0.016 | 4.75 | < 0.001 |
|  |  | Cut × Forb Rich | | 0.005 | 0.015 | 0.319 | 0.75 |
|  |  | Random effects: | | σ | SD | Obs. | Sites |
|  |  |  | Site identity | 0.577 | 0.759 | 57 | 19 |
| Bee abundance (*Excluding cleptoparasites)* | | | | β | SE | z | P |
|  |  | Intercept (Uncut) | | 0.083 | 0.427 | 0.195 | 0.846 |
|  |  | Cut-Remove | | -0.268 | 0.238 | -1.129 | 0.259 |
|  |  | Cut | | 0.326 | 0.235 | 1.388 | 0.165 |
|  |  | Forb Rich | | -0.019 | 0.014 | -1.310 | 0.190 |
|  |  | Elevation | | -0.003 | 0.001 | -2.235 | 0.025 |
|  |  | Cut-Remove × Forb Rich | | 0.070 | 0.016 | 4.342 | 0.000 |
|  |  | Cut × Forb Rich | | 0.004 | 0.015 | 0.252 | 0.801 |
|  |  | Random effects: | | σ | SD | Obs. | Sites |
|  |  |  | Site identity | 0.585 | 0.765 | 57 | 19 |
