# Supplementary material for: The effects of habitat management on the species, phylogenetic and functional diversity of bees are modified by the environmental context
Source: Ecol Evol. 2016 Jan 18;6(4):961–73. doi: 10.1002/ece3.1963 (PMC4761776; doi:10.1002/ece3.1963)
Supplement: Supplementary file 5 — Table S5. Backward elimination of variables and final model outputs from analyses of phylogenetic diversity. [file ECE3-6-0961-s005.docx]

Table S5.1 Backward elimination of variables from the full models. Response variables were: The phylogenetic species richness (PSR), the phylogenetic species variability (PSV), the phylogenetic species evenness (PSE) and the phylogenetic species clustering (PSC). The variables in the full models were: Treatment type, Forb species richness, Elevation, Treatment type × Forb species richness, Treatment type × Elevation, Forb species richness × Elevation and the log(Number of Trap months) was included as an offset variable. In the first step (step 1) the full model was tested against a full model where the offset variable was excluded by comparing the deviance statistics between the models using χ^2^ tests. The offset variable was included if it decreased the deviance, and dropped if it increased the deviance. Variables with p-values > 0.05 were dropped from the model. Outputs from the final models are shown in table S5.2. Note that for the PSR the interaction term Treatment type × Elevation was included in the analyses, despite not being significant at the 0.05 level, since its inclusion significantly improved the model fit (χ^2^ = 8.44, p < 0.038) compared to a model where Treatment type × Elevation and Elevation (df = 1, LRT = 2.572, p = 0.109) were removed.

| Response | Explanatory and offset variable | LRT for variable | step |
| --- | --- | --- | --- |
| PSR | Offset variable: *included* | χ^2^ = 1.27, p < 0.001 | 1 |
|  | Forb species richness × Elevation | df = 1, LRT = 0.013, p = 0.909 | 2 |
|  | Treatment type × Elevation | df = 2, LRT = 5.864, p = 0.053 | 3* |
|  | Elevation | df = 1, LRT = 2.572, p = 0.109 | 4 |
| PSV | Offset variable: *Dropped* | χ^2^ = 24.402, p < 0.001 | 1 |
|  | Treatment type × Forb species richness | df = 2, LRT = 1.269, p = 0.530 | 2 |
|  | Forb species richness × Elevation | df = 1, LRT = 0.444, p = 0.505 | 3 |
|  | Treatment type × Elevation | df = 2, LRT = 1.568, p = 0.457 | 4 |
|  | Elevation | df= 1, LRT = 0.003, p = 0.955 | 5 |
|  | Treatment type | df = 2, LRT = 1.522, p = 0.467 | 6 |
|  | Forb species richness | df = 1, LRT = 2.533, p = 0.112 | 7 |
| PSE | Offset variable: *Dropped* | χ^2^ = 18.76, p < 0.001 | 1 |
|  | Treatment type × Elevation | df = 2, LRT = 0.154, p = 0.912 | 2 |
|  | Forb species richness × Elevation | df = 1, LRT = 0.682, p = 0.409 | 3 |
|  | Treatment type × Forb species richness | df = 2, LRT = 2.252, p = 0.324 | 4 |
|  | Forb species richness | df = 1, LRT = 0.261, p = 0.609 | 5 |
|  | Treatment type | df = 2, LRT = 3.835, p = 0.147 | 6 |
|  | Elevation | df = 1, LRT = 2.189, p = 0.139 | 7 |
| PSC | Offset variable: *Dropped* | χ^2^ = 17.007, p < 0.001 | 1 |
|  | Treatment type × Elevation | df = 2, LRT = 1.864, p = 0.394 | 2 |

Table S5.2. The effects of experimental treatment and habitat conditions on the phylogenetic diversity of bees sampled in treatment plots. The experimental treatments mimicked three different management practices (Uncut, Cut, and Cut-Remove, Fig. 1). Forb Rich = the species richness of forbs within the treatment plot. The Phylogenetic Species Richness and the difference between the species richness and PSR were fitted using generalized linear mixed effects models (GLMM) with Gamma distributions and log-link functions. The Phylogenetic species clustering was fitted with linear mixed models (See main text for Likelihood test statistics).

| Phylogenetic Species Richness (PSR) | | | | | | | β |  | SE |  | t |  | p |
| --- | --- | --- | --- | --- | --- | --- | --- | --- | --- | --- | --- | --- | --- |
|  |  | | Intercept (Uncut) | | |  | -1.060 |  | 0.328 |  | -3.230 |  | 0.001 |
|  |  | | Cut-Remove | | |  | -0.008 |  | 0.240 |  | -0.034 |  | 0.973 |
|  |  | | Cut | | |  | 0.462 |  | 0.264 |  | 1.754 |  | 0.079 |
|  |  | | Forb Rich | | |  | -0.032 |  | 0.012 |  | -2.576 |  | 0.010 |
|  |  | | Elevation | | |  | <0.001 |  | 0.001 |  | 0.126 |  | 0.900 |
|  |  | | Cut-Remove × Forb Rich | | |  | 0.082 |  | 0.014 |  | 5.855 |  | <0.001 |
|  |  | | Cut × Forb Rich | | |  | 0.034 |  | 0.013 |  | 2.627 |  | 0.009 |
|  |  | | Cut-Remove × Elevation | | |  | -0.002 |  | 0.001 |  | -2.612 |  | 0.009 |
|  |  | | Cut × Elevation | | |  | -0.002 |  | 0.001 |  | -1.858 |  | 0.063 |
|  | Random effects: | | | | |  | σ |  | SD |  | Obs. |  | Sites |
|  |  | |  | | Site identity |  | 0.113 |  | 0.336 |  | 46 |  | 18 |
|  |  | |  | | Residual |  | 0.099 |  | 0.315 |  |  |  |  |
| Phylogenetic species clustering (PSC) | | | | | | | β |  | SE |  | t |  |  |
|  |  |  | | Intercept (Uncut) | |  | 0,407 |  | 0,073 |  | 5,616 |  |  |
|  |  |  | | Forb Rich | |  | -0,009 |  | 0,006 |  | -1,480 |  |  |
|  |  |  | | Cut-Remove | |  | -0,084 |  | 0,071 |  | -1,179 |  |  |
|  |  |  | | Cut | |  | -0,058 |  | 0,076 |  | -0,759 |  |  |
|  |  |  | | Elevation | |  | -0,001 |  | 0,0003 |  | -1,937 |  |  |
|  |  |  | | Cut-Remove × Forb Rich | |  | 0,013 |  | 0,005 |  | 2,525 |  |  |
|  |  |  | | Cut × Forb Rich | |  | 0,010 |  | 0,005 |  | 1,812 |  |  |
|  |  |  | | Forb rich × Elevation | |  | 3E-05 |  | 1E-05 |  | 2.020 |  |  |
|  |  | Random effects: | | | |  | σ |  | SD |  | Obs. |  | Sites |
|  |  |  | | Site | |  | 0.0004 |  | 0.020 |  | 46 |  | 18 |
|  |  |  | | Residuals | |  | 0.012 |  | 0.110 |  |  |  |  |
