# Supplementary material for: The effects of habitat management on the species, phylogenetic and functional diversity of bees are modified by the environmental context
Source: Ecol Evol. 2016 Jan 18;6(4):961–73. doi: 10.1002/ece3.1963 (PMC4761776; doi:10.1002/ece3.1963)
Supplement: Supplementary file 6 — Table S6. Backward elimination of variables and final model outputs from analyses of functional diversity. [file ECE3-6-0961-s006.docx]

Table S6.1 Backward elimination of variables from the full models. Response variables were: The functional species richness (FSR), the functional dispersion (FDis), the functional evenness (FEve), the community weighted mean body size (ITD), the proportion of below ground nesters (Below) and the proportion of pollen specialists (Oligo.). The variables in the full models were: Treatment type, Forb species richness, Elevation, Treatment type × Forb species richness, Treatment type × Elevation, Forb species richness × Elevation and the log(Number of Trap months) was included as an offset variable. In the first step (step 1) the full model was tested against a full model where the offset variable was excluded by comparing the deviance statistics between the models using χ^2^ tests. The offset variable was included if it decreased the deviance , and dropped if that increased the deviance. Variables with p-values > 0.05 were dropped from the model. Outputs from the final models are shown in table S6.2.

| Response | | variable | LRT for variable | step |
| --- | --- | --- | --- | --- |
| FSR | | Offset variable: *included* | χ^2^ = 1.67, p < 0.001 | 1 |
|  |  | Treatment type × Elevation | df = 2, LRT = 0.37, p = 0.83 | 2 |
| FDis | | Offset variable: *Dropped* | χ^2^ = 28.79, p < 0.001 | 1 |
|  |  | Treatment type × Forb species richness | df = 2, LRT = 0.959, p = 0.619 | 2 |
|  |  | Treatment type × Elevation | df = 2, LRT = 1.688, p = 0.430 | 3 |
|  |  | Forb species richness × Elevation | df = 1, LRT = 1.599, p = 0.206 | 4 |
|  |  | Elevation | df = 1, LRT = 2.622, p = 0.105 | 5 |
|  |  | Forb species richness | df = 1, LRT = 2.766, p = 0.096 | 6 |
|  |  | Treatment type | df = 1, LRT = 3.806, p = 0.149 | 7 |
| FEve | | Offset variable: *Dropped* | χ^2^ = 4.00, p < 0.001 | 1 |
|  |  | Forb species richness × Elevation | df = 1, LRT = 0.229, p = 0.632 | 2 |
|  |  | Treatment type × Elevation | df = 1, LRT = 0.770, p = 0.680 | 3 |
|  |  | Treatment type × Forb species richness | df = 2, LRT = 1.486, p = 0.476 | 4 |
|  |  | Treatment type | df = 2, LRT = 2.523, p = 0.283 | 5 |
| ITD | | Offset variable: *Dropped* | χ^2^ = 1.502, P < 0.001 | 1 |
|  |  | Treatment type × Forb species richness | df = 2, LRT = 1.185, p = 0.553 | 1 |
|  |  | Treatment type × Elevation | df = 2, LRT = 1.408, p = 0.495 | 2 |
|  |  | Treatment type | df = 2, LRT = 1.269, p = 0.53 | 3 |
|  |  | Forb species richness × Elevation | df = 1, LRT = 0.352, p = 0.553 | 4 |
| % Below | | Offset variable: *Not included* | NA | 1 |
|  | | Treatment type × Elevation | df = 2, LRT = 0.049, p = 0.976 | 2 |
|  | | Forb species richness × Elevation | df = 2, LRT = 1.206, p = 0.272 | 3 |
|  | | Elevation | df = 1, LRT = 0.379, p = 0.538 | 4 |
|  | | Treatment type × Forb species richness | df = 2, LRT = 5.037, p = 0.081 | 5 |
|  | | Forb species richness | df = 2, LRT = 0.003, p = 0.958 | 6 |
| % Oligo. | | Offset variable: *Not included* | NA | 1 |
|  | | Forb species richness × Elevation | df = 1, LRT = 3.355, p = 0.067 | 2 |
| Emergence | | Offset variable: *included* | Df = 12, χ^2^ = 0.379, p < 0.001 | 1 |
|  | | Treatment type × Forb species richness | Df = 2, LRT = 0.432, p = 0.806 | 2 |
|  | | Forb species richness × Elevation | Df = 1, LRT = 2.37, p = 0.123 | 3 |

Table S6.2 Outputs from final mixed effect models on the functional diversity within treatment plots. See Table 3 for LRT test statistics and text for details.

|  | Functionally singular species richness (FSSR) | | | β | |  | | SE | |  | | z | |  | | p | |  |
| --- | --- | --- | --- | --- | --- | --- | --- | --- | --- | --- | --- | --- | --- | --- | --- | --- | --- | --- |
|  |  | Intercept (Uncut) | | -1.188 | |  | | 0.212 | |  | | -5.593 | |  | | <0.001 | |  |
|  |  | Forb species richness | | -0.012 | |  | | 0.018 | |  | | -0.688 | |  | | 0.491 | |  |
|  |  | Cut-Remove | | -0.355 | |  | | 0.296 | |  | | -1.199 | |  | | 0.231 | |  |
|  |  | Cut | | 0.173 | |  | | 0.292 | |  | | 0.591 | |  | | 0.554 | |  |
|  |  | Elevation (standardized to achieve convergence) | | -0.522 | |  | | 0.165 | |  | | -3.161 | |  | | 0.002 | |  |
|  |  | Cut-Remove × Forb species richness | | 0.077 | |  | | 0.023 | |  | | 3.352 | |  | | 0.001 | |  |
|  |  | Cut × Forb species richness | | 0.023 | |  | | 0.022 | |  | | 1.015 | |  | | 0.310 | |  |
|  |  | Forb species richness × Elevation (standardized) | | 0.033 | |  | | 0.013 | |  | | 2.494 | |  | | 0.013 | |  |
|  |  | Random effects: | | σ | |  | | SD | |  | | Obs. | |  | | Sites | |  |
|  |  |  | Site identity | 0.071 | |  | | 0.266 | |  | | 55 | |  | | 19 | |  |
|  | Functional evenness (FEve) | | | β | |  | | SE | |  | | z | |  | |  | |  |
|  |  | Intercept (Uncut) | | | 0.749 | |  | | 0.058 | |  | | 13.008 | |  | |  | |
|  |  | Forb species richness | | | -0.012 | |  | | 0.003 | |  | | -3.937 | |  | |  | |
|  |  | Elevation | | | 0.0004 | |  | | 0.0002 | |  | | 1.997 | |  | |  | |
|  |  | Random effects: | | | σ | |  | | SD | |  | | Obs. | |  | | Sites | |
|  |  |  | Site identity | | 0.001 | |  | | 0.023 | |  | | 40 | |  | | 17 | |
|  |  |  | Residuals | | 0.018 | |  | | 0.134 | |  | |  | |  | |  | |
|  | Mean body size of bee individuals (ITD) | | | | β | |  | | SE | |  | | t | |  | |  | |
|  |  | Intercept | | | 2.067 | |  | | 0.157 | |  | | 13.136 | |  | |  | |
|  |  | Forb species richness | | | -0.026 | |  | | 0.007 | |  | | -3.528 | |  | |  | |
|  |  | Elevation | | | 0.001 | |  | | 0.0004 | |  | | 3.087 | |  | |  | |
|  |  | Random effects | | | σ | |  | | SD | |  | | Obs. | |  | | Sites | |
|  |  |  | Site | | 0.034 | |  | | 0.184 | |  | | 55 | |  | | 19 | |
|  |  |  | Residual | | 0.092 | |  | | 0.304 | |  | |  | |  | |  | |
|  | Proportion of below ground nesters | | | | β | |  | | SE | |  | | z | |  | | p | |
|  |  | Intercept (Uncut) | | | 2.413 | |  | | 0.350 | |  | | 6.888 | |  | | <0.001 | |
|  |  | Cut-Remove | | | -1.358 | |  | | 0.356 | |  | | -3.811 | |  | | <0.001 | |
|  |  | Cut | | | -1.109 | |  | | 0.370 | |  | | -2.997 | |  | | 0.003 | |
|  |  | Random effects | | | σ | |  | | SD | |  | | Obs. | |  | | Sites | |
|  |  |  | Site | | 0.269 | |  | | 0.518 | |  | | 55 | |  | | 19 | |
|  | Proportion of Oligolectic individuals | | | | β | |  | | SE | |  | | z | |  | | p | |
|  |  | Intercept (Uncut) | | | -1.451 | |  | | 0.766 | |  | | -1.896 | |  | | 0.058 | |
|  |  | Cut-Remove | | | 1.327 | |  | | 0.810 | |  | | 1.638 | |  | | 0.101 | |
|  |  | Cut | | | -0.036 | |  | | 0.793 | |  | | -0.046 | |  | | 0.963 | |
|  |  | Forb species richness | | | -0.199 | |  | | 0.059 | |  | | -3.357 | |  | | 0.001 | |
|  |  | Elevation | | | 0.012 | |  | | 0.004 | |  | | 3.026 | |  | | 0.002 | |
|  |  | Cut-Remove × Forb species richness | | | 0.133 | |  | | 0.059 | |  | | 2.266 | |  | | 0.023 | |
|  |  | Cut × Forb species richness | | | 0.165 | |  | | 0.057 | |  | | 2.893 | |  | | 0.004 | |
|  |  | Cut-Remove × Elevation | | | -0.011 | |  | | 0.004 | |  | | -2.729 | |  | | 0.006 | |
|  |  | Cut × Elevation | | | -0.008 | |  | | 0.004 | |  | | -1.926 | |  | | 0.054 | |
|  |  | Random effects | | | σ | |  | | SD | |  | | Obs. | |  | | Sites | |
|  |  |  | Site identity | | 0.412 | |  | | 0.642 | |  | | 55 | |  | | 19 | |
|  | CWM emergence time | | | | β | |  | | SE | |  | | t | |  | |  | |
|  |  | Intercept (Uncut) | | | -1,288 | |  | | 0,356 | |  | | -3,624 | |  | |  | |
|  |  | Cut-Remove | | | 0,867 | |  | | 0,336 | |  | | 2,582 | |  | |  | |
|  |  | Cut | | | 0,855 | |  | | 0,335 | |  | | 2,551 | |  | |  | |
|  |  | Forb species richness | | | -0,029 | |  | | 0,014 | |  | | -2,125 | |  | |  | |
|  |  | Elevation | | | 0,002 | |  | | 0,001 | |  | | 2,012 | |  | |  | |
|  |  | Cut-Remove × Elevation | | | -0,003 | |  | | 0,001 | |  | | -2,736 | |  | |  | |
|  |  | Cut × Elevation | | | -0,004 | |  | | 0,001 | |  | | -3,108 | |  | |  | |
|  |  | Random effects | | | σ | |  | | SD | |  | | Obs. | |  | | Sites | |
|  |  |  | Site identity | | 0.210 | |  | | 0.458 | |  | | 55 | |  | | 19 | |
|  |  |  | Residual | | 0.218 | |  | | 0.467 | |  | |  | |  | |  | |
